# Investigating the effect of materials and structures for negative pressure ventilators suitable for pandemic situation

**DOI:** 10.1007/s42247-021-00181-x

**Published:** 2021-04-01

**Authors:** Nada Abughanam, Shahd Sameer Mohammed Gaben, Muhammad E. H. Chowdhury, Amith Khandakar

**Affiliations:** Department of Electrical Engineering, Qatar University, Doha, 2713 Qatar

**Keywords:** Mechanical ventilators, Positive pressure ventilator, Negative pressure ventilator, ANSYS simulation

## Abstract

The onset of the corona virus disease 2019 (COVID-19) pandemic caused shortages in mechanical ventilators (MVs) essential for the intensive care unit (ICU) in the hospitals. The increasing crisis prompted the investigation of ventilators which is low cost and offers lower health complications. Many researchers are revisiting the use of negative pressure ventilators (NPVs), due to the cost and complications of positive pressure ventilators (PPVs). This paper summarizes the evolution of the MVs, highlighting the limitations of popular positive and negative pressure ventilators and how NPV can be a cost-effective and lower health complication solution. This paper also provides a detailed investigation of the structure and material for the patient enclosure that can be used for a cost-effective NPV system using ANSYS simulations. The simulation results can confirm the selection and also help in developing a low cost while based on readily available materials. This can help the manufacturer to develop low-cost NPV and reduce the pressure on the healthcare system for any pandemic situation similar to COVID-19.

## Introduction

Wars, natural disasters, pandemics, and accidents all require handling mass casualty situations which have been found problematic over the years. Many victims could require lung ventilation for survival, which makes the presence of ventilators and experienced personnel the center of preparedness for such situations. Thus, the absence of mechanical ventilators (MVs) and experienced rescuers can lead to the mortality of victims with respiratory failure. In the 1950s, at the onset of the polio epidemic, there was a serious shortage of ventilators, which ushered in the creation of a new type of ventilation to satisfy the demand [1]. A similar situation is witnessed now; in addition to patients afflicted with respiratory illnesses and injuries, 80 million global cases of corona virus of 2019 (COVID-19) have been recorded up to this moment, where hundreds of thousands of ventilators are needed [2]. According to the Center for Disease Control (CDC), fast mass production of MVs has become a necessity to compensate for the supply shortage in many countries as COVID-19 outbreaks. While ventilators are highly demanded due to the outbreak of COVID-19, many of the current ventilators are used by patients for other respiratory diseases. Millions of people are affected by lung diseases every year, where approximately 65 million people are afflicted with chronic obstructive pulmonary disease (COPD) with 3 million deaths per year, in addition to 4 million lives lost due to lower respiratory tract infections and 1.6 million deaths due to lung cancer [3].

**Fig. 1:**
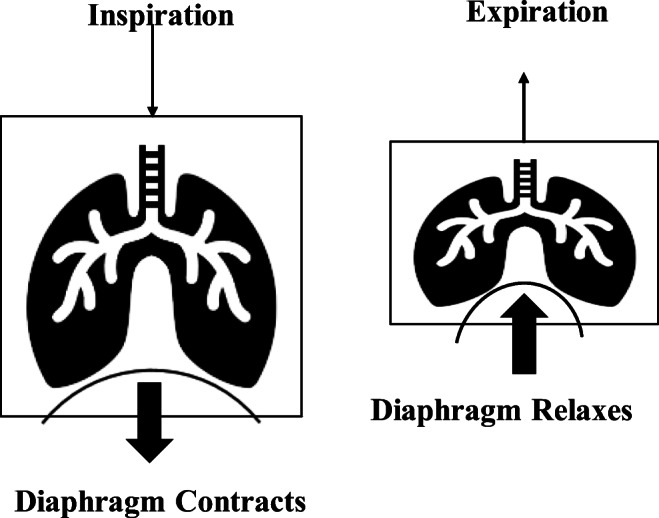
Changes in the lungs during inspiration and expiration

Before the discussion on the MVs, it would be helpful to understand the pulmonary ventilation process. It is a cycle that consists of two phases, inspiration and expiration, and the pressure, flow, and volume of the organs change during each of them. Pulmonary ventilation occurs due to the difference in pressure in the atmosphere and the lungs (i.e., transpulmonary pressure). The diaphragm contracts during inspiration, increasing the volume of the chest cavity which creates a negative intrapleural pressure that expands the lungs and decreases the pressure inside them. As gasses move from areas with higher pressure to areas with lower pressure, the decrease in pressure causes the air outside to rush into the lungs. Similarly, the diaphragm relaxes during expiration and the volume of the chest cavity decreases due to the elastic recoil. This increases the pressure in the lungs, and the air is pushed out of the lungs into the atmosphere (refer to Fig. 1). The thoracic cavity also changes during the inspiration and expiration.

During the COVID-19 pandemic, the hospitals were overburdened with patients and were facing a shortage of necessary medical equipment such as ventilators, which lead to a lot of research in low-cost and low-risk ventilator’s design [4–7]. MIT engineers created an open-source project, based on a manual Ambu bag-valve-mask that could be shared and reconstructed worldwide [8]. In the case of mandatory ventilation when intensive care/anesthesia ventilators are unavailable, a modified noninvasive ventilation (NIV) device was shown to provide positive pressure ventilation [9]. A low-cost pneumatic-type PPV using three electrically controlled solenoid valves, a pressure chamber, the patient breathing circuit, a positive end-expiratory pressure valve, and an electronics control system was proposed in [10]. A CRISIS ventilator using 3D printing technology was developed with two dials (pressure and respiration rate) for COVID-19 crisis time [11]. A bilevel positive airway pressure (BiPAP) machine from a device used for sleep apnea was modified to an emergency ventilator that can help COVID-19 patients using 3-D printing [12]. Daoud et al. [13] reported a remote-controlled ventilator for the ICU to avoid the risk of the highly infectious virus of the medical personnel. Islam et al. in [14] have discussed different wearable monitoring devices and respiratory medical equipment such as ventilators during the COVID-19 pandemic making a special mention of the open-source positive pressure ventilation device (OSPPVD). Islam et al. in [15] have provided a brief overview and comparison between the different breathing aid devices such as ventilators and concluded with future directions in terms of affordable technologies. In a study from Stanford University and funded by ChanZukerberg BioHub, Raymond et al. [16] have introduced a proof of concept low-cost ventilator design following the emergence of use guidance by the US Food and Drug Administration (FDA) to allow a patient to be treated and capable of supporting different treatment paradigms. Horvath et al. [17] developed an innovative and effective pedagogical organosynthetic soft robotic respiratory simulator tool which can be used for educating students on respiratory physiology and pathology in a user-controlled, interactive manner. Mirvakili et al. [18] demonstrated a low-cost, portable volume-controlled mechanical ventilator using pneumatic artificial muscles. Darwood et al. [19] proposed a portable positive pressure mechanical ventilation at a reduced cost, while autonomously can monitor patient condition and important safety parameters. A prototype ventilator was constructed and evaluated using an anesthetic test-lung as a patient surrogate. The OneBreath ventilator, a full-featured ventilator intended for hospital and pre-hospital use, is proposed for low-resource environments, novice users, and adult and pediatric patients. A simpler and less expensive design architecture, with a cost of goods that is a fraction of currently available ventilators but that provides standard-of-care performance and features, was the core objective of this PPV [20]. A review of several open-source ventilators for COVID-19 was reported in [4].

The process of mechanical ventilation started five centuries ago when Professor of Anatomy Andreas Vesalius published a paper on anatomy in 1543 that has the first known mentioning of positive pressure ventilation. It was mentioned that a tube was inserted into the trachea which was then blown to make an animal return to life and breathe again, similar to the tracheostomy procedure that is performed today [21]. In the centuries after, mouth-to-mouth resuscitation was widely used, until the nineteenth century when mechanical ventilation came into the scene. In 1832, the first tank ventilator was invented by Scottish John Dalziel in which a patient sat upright in a box with their head outside the box, where sub-atmospheric pressure was created around the body using bellows inside the box that increased and decreased the pressure.

**Fig. 2:**
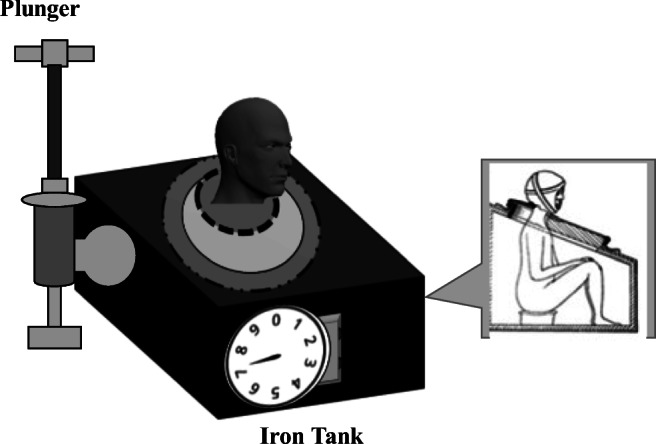
First American tank ventilator

The first American tank ventilator was created in 1864 by Alfred Jones, the tank similarly enclosed the patient from the neck down but the negative pressure was created using a plunger (Fig. 2). The first cuirass was created by Ignez von Hauke in 1874 in Austria, and he later made a tank ventilator when he found the cuirass unsuitable for agitated patients [22].

**Fig. 3:**
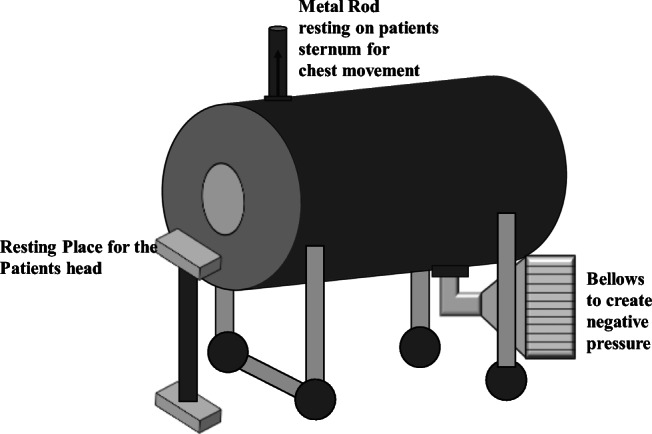
The Spirophore

The first iron lung, named the Spirophore (Fig. 3), was built in 1876 by the French Eugene Joseph Woillez. The Spirophore was a metal cylinder that surrounded the body and had a rubber diaphragm seal around the neck with bellows outside the cylinder creating negative pressure. It had a metal rod that rested on the patient’s sternum to measure the movement of the chest. Many other negative pressure ventilators continued to be made throughout the years, such as the tank ventilator in 1887 by Charles Breuillard, the jacket ventilator by Alexander Graham Bell, the portable cuirass ventilator by Rudolph Eisenmenger in 1901, and the giant negative pressure room by Ernst Ferdinand Sauerbruch in 1904, to allow a surgeon to operate on the patient which previous designs did not allow, and many more [23].

**Fig. 4:**
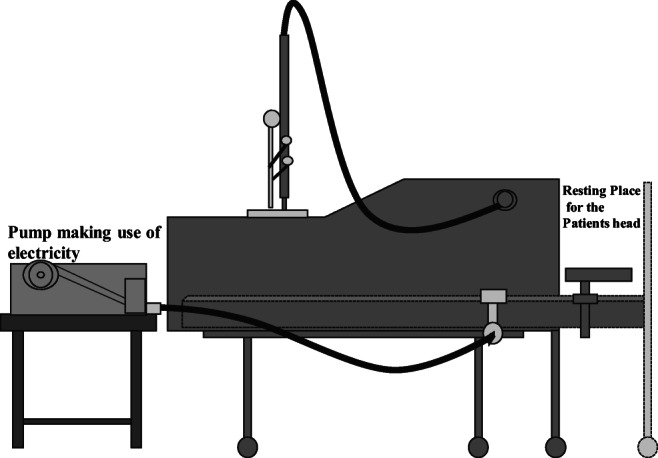
Drinker and Shaw’s iron lung

In 1916, there was a resurgence of poliomyelitis epidemics that caused many ventilator innovations. However, only the iron lung made by Drinker and Shaw in Boston in 1929 gained widespread use (Fig. 4). The tank was a metal cylinder with one end with an airtight collar and the other with a piston pump that changed the pressure inside the tank. The previous ventilators all had in common, i.e., manual operation was needed to create pressure changes; Drinker and Shaw’s iron lung however was able to use a pump due to the availability of reliable electricity [23].

The peak of the polio epidemic was reached in 1931 when John Haven Emerson modified the iron lung due to it being cumbersome, complex, bulky, and expensive. He simplified the design, cut its cost in half, and added several improvements such as portholes and the ability to operate the pump manually should a power failure occur. In 1937, the Both Respirator was invented by Edward and Donald Both. It was lighter, cheaper, and easier to transport, owing to the fact it was made of plywood and was extensively used in the UK.

The continued danger from polio inspired the creation and modification of more tank, jacket, and cuirass ventilators. That was until the polio epidemic of 1952 hit Denmark, where 37% of the 866 paralytic patients required ventilation. The shortage of negative pressure ventilators prompted Bjorn Ibsen to propose to Lassen to use tracheostomy to deliver positive pressure by squeezing a bag connected to the tube, which was previously only used during surgery briefly. It was observed that the previous mortality rate of 80% using the cuirass fell to about 40% using positive pressure [23].

The focus then shifted from ventilator support to improving or maintaining gas exchange, where negative pressure ventilators were shown to be much less effective than positive pressure ventilators, leading to the increased use of positive pressure ventilators. It can be seen that the use of positive pressure ventilation dominated compared to negative pressure ventilation, and it has witnessed many technical improvements over the years.

Many of the advancements made are to reduce the side effects that are caused by positive pressure ventilation, although negative pressure ventilation has some side effects too. The complications of negative pressure ventilation include the absence of upper airway protection which might cause aspiration. Moreover, there is a possibility of upper airway obstruction in unconscious patients and patients with sleep apnea. Besides, as negative pressure ventilators restrict motion, back pain is a common issue in addition to rib fractures. However, these complications seem minor when compared with those resulting from positive pressure ventilation.

There are many complications associated with positive pressure ventilation, although those resulting from invasive positive pressure ventilation are more than those associated with non-invasive positive pressure ventilation. Invasive positive pressure ventilation complications include ventilator-associated lung injury and barotrauma that result from high inspiratory pressure, ventilator-associated pneumonia due to the endotracheal tube providing direct contact between the world and the lower respiratory tract that bacteria can invade, oxygen toxicity, and neuromuscular complications that can result from the patient’s need for sedation or paralysis and might cause the diaphragm to atrophy, making it difficult for patients to be weaned off ventilators [24]. Complications associated with non-invasive positive pressure ventilation are less severe in comparison and include conjunctival irritation that is caused by air leaking from under the mask into the sinuses and eyes. Air leakage through the mouth can also cause oral or nasal dryness. Other common complaints include nasal congestion, aspiration, and gastric insufflation, in addition to nasal pain and ulceration that result from the mask pressure [25].

It is also worth noting concern is that placing a patient on positive pressure ventilation can affect the heart-lung physiology and their hemodynamic status, due to their natural negative pressure ventilation being changed to positive pressure [26]. This issue can be avoided if negative pressure ventilation is used. However, it is known that negative pressure ventilation has its own set of issues that prevented it from being widespread. It was bulky and expensive, and it also hindered the accessibility and actions of the clinicians.

Keeping this in mind, a modified negative pressure ventilator can be proposed, so that it can be developed and used aside from the PPV and help the existing medical infrastructure to cope with the huge number of patients during pandemics. Following the iron lung design, the length of the tank can be lessened to reach the hips, which gives the clinicians access to the patient’s lower body without reducing the effectiveness since the lower body does not participate in the respiration process. This also means that it will be less heavy and will take less space. For it to be at the same level as modern positive pressure ventilators, a sophisticated control system must be designed to allow easy control of the patient’s pressure, respiratory rate, and inspiration/expiration (I:E) ratio, in addition to alarms that alert the clinicians to any unexpected occurrences.

Before any additional modifications, however, it is necessary to first examine the effectiveness of the structure of the patient enclosure that was used in the iron lung, and the possibility of finding a more effective shape that can deliver respiratory support more effectively. Besides, the best material to implement the structure should be closely examined. Thus, this manuscript (i) summarizes the evolution of the MVs, (ii) highlights the limitations of popular positive pressure ventilators (PPVs) and negative pressure ventilators (NPVs) and how NPV can be a cost-effective and lower health complication solution, (iii) describes the structure and material that can be used for the patient enclosure of an NPV, and (iv) simulated results of the different structures and materials. The design process is optimized to develop a lighter and thinner ventilator with low-cost design materials and the most efficient design that will require less support, as the current ventilators are really expensive, as seen in the literature above. The health complications are way lesser in NPV as they go along the natural breathing process rather than go against it as in PPV.

Thus, the rest of the paper is divided into the following sections: Section 2 provides the methodology of the whole work along with the discussion of the advantages and limitations of the structural design of MVs, followed by Section 3 which describes some modern NPVs. Sections 4 and 5 elaborate on the design and material for NPVs in detail and Section 6 provides the simulation results. Finally, the conclusion and future work is provided in Section 7.

## Advantages and limitations

**Fig. 5:**
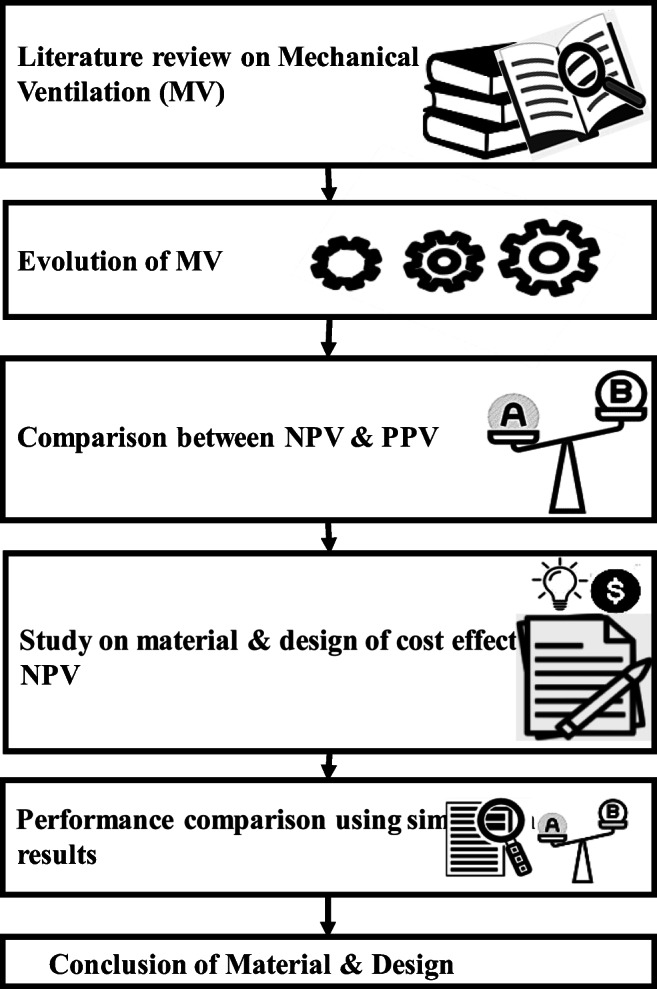
Methodology adopted in the paper

The methodology adopted in the paper can be seen in Fig. 5 where the authors have done an extensive literature review on MV to extract some useful designs from the past and current times. The authors have shown the evolution of the MVs already in Section 1. Then, the authors have provided the comparison between the popular PPV and existing NPV solutions. The authors have investigated the various materials and design can work effectively and confirm it using simulation results. Finally, the conclusion is provided which can be added to the existing body of knowledge and will also be used by the authors in the future implementation and testing of such a design.

Mechanical ventilation artificially creates the pressure difference that leads to ventilation. Positive pressure ventilation differs from negative pressure ventilation in the way pressure difference is created. In positive pressure ventilation, the air is pushed into the lungs, and the alveolar pressure increases, expanding the lungs where the elastic recoil allows exhalation. Meanwhile, in negative pressure ventilation, negative pressure is developed around the chest and abdomen which expands the lungs, where the intrapleural and alveolar pressures decrease which makes airflow into the lungs and alveoli. Exhalation occurs due to the elastic recoil when the pressure around the chest is back to ambient pressure [27]. Owing to the different methods in which negative pressure ventilation and positive pressure ventilation provide ventilator support to patients, several techniques to apply each were used.

To begin with, in all its types, negative pressure ventilation requires the chest and abdomen to be enclosed in an airtight chamber. These types include tank ventilators, jacket ventilators, and cuirasses [28]. Tank ventilators surround the entire body of the patient from the neck down, with an airtight rubber seal around the neck in addition to windows and portholes that allowed observations. On the other hand, jacket ventilators consist of a garment that ends below the hips and is suspended over a framework made of plastic or metal grids. In jacket ventilators, the expansion of the chest and abdomen is not limited, but it is cold to wear due to leaks and awkward to put on. Finally, the cuirass is a shell with edges padded with airtight material where the proper fitting is difficult with standard-sized shells; they are also the least effective.

On the other hand, there are two main methods to provide positive pressure ventilation: invasive positive pressure ventilation and non-invasive positive pressure ventilation. Invasive positive pressure ventilation is provided to a sedated or paralyzed patient via an endotracheal tube placed in the patient’s airway or a tracheostomy tube that creates an artificial airway. Meanwhile, non-invasive positive pressure ventilation is provided via a full face mask, nasal mask, oronasal mask, nasal pillows, and mouthpieces. It also does not require the patient to be sedated, unlike invasive positive pressure ventilation. In non-invasive positive pressure ventilation, continuous positive airway pressure (CPAP), which provides a constant positive pressure, and bilevel positive airway pressure (BiPAP), which provides a baseline pressure and a higher pressure for when the patient tries to inspire, are the two most common modes [24].

Due to its method of delivery, although positive pressure ventilation has saved countless lives ever since its widespread use decades ago, it is still associated with many complications that can adversely affect the patient’s health. Some of these complications have been mentioned since 1744 when John Fothergill mentioned in an essay William Tossach’s publication wherein he applied mouth-to-mouth resuscitation on a pulseless coalminer. It was mentioned how the air out of a person’s mouth does not have the possibility of harming the other person, while bellows cannot guarantee that [21].

One of these complications is barotrauma, where the alveoli rupture due to the high pressure entering the lungs. This can cause pneumothorax, pneumoperitoneum, and subcutaneous emphysema, all of which have a high mortality rate. Besides, several extreme complications associated with invasive positive pressure ventilation arise from the act of intubation itself. During endotracheal intubation, a tube is inserted into the patient’s airway through the mouth. This insertion has hemodynamic effects and results in a decreased cardiac output, where the right-ventricular preload is decreased and pulmonary vascular resistance is increased, both of which may decrease cardiac output [29]. When inserted into the patient’s airway, the endotracheal tube occupies the free space allocated for the expansion of the lungs. Therefore, as the lungs expand during mechanical breathing, limited space is left for the heart, ending up being crushed and resulting in a decreased cardiac output.

Another problem that arises from inserting an endotracheal tube into the patient’s airway is the high risk of infection and ventilator-associated pneumonia. This is because of connecting the respiratory tract to the outside environment via intubation and the difficulty of disinfecting the airway properly. Thus, patients are at a higher risk of respiratory infections due to the colonization of the endotracheal tube in the airway and the difficulty of secretion clearance [21]. This is expected since normal airway hygiene happens through coughing, which is not possible with an endotracheal tube inserted into the patient’s airway. Moreover, with normal airway hygiene being impossible, clinicians use suctioning to clear the airway. However, the act of suctioning can cause several complications to the lungs, where patients are at a higher risk of atelectasis, pneumonia, and respiratory failure due to the impairment of secretion clearance [30].

Sedation-related complication is another type of complications associated with invasive positive pressure ventilation. When patients are moved from noninvasive positive pressure ventilation to invasive positive pressure ventilation, they require sedation or sometimes temporary paralysis for them to tolerate a tube placed down their airway. Patients who have ventilator desynchrony also require to be paralyzed. This paralytic medication can cause neuromuscular complications that can lead to the atrophy of the diaphragm such as the depletion of bioenergetic neuron reserves and the increase of inflammatory cytokines [24].

Complications of prolonged weaning are other complications that can arise from sedating patients under invasive positive pressure ventilation. Weaning patients from positive pressure ventilation, generally, requires them to be conscious and to have close to normal respiratory functioning. Weaning patients from invasive positive pressure ventilation at the right time is very essential and any delays in the process will cause an increase in invasive positive pressure ventilation complications, a longer stay in the hospital, and a higher mortality rate [31]. Thus, sedation here is not a direct contributor to those complications, but rather it causes longer stays under mechanical ventilation which in turn increases the risk of all other complications associated with it.

This is in addition to problems that result from noninvasive positive pressure ventilation that include discomfort or ulcers and lacerations due to the interface by which noninvasive positive pressure ventilation is applied.

The final set of complications associated with positive pressure ventilation is ventilation mismanagement complications. Throughout history, positive pressure ventilators have evolved enormously until reaching the fourth generation, currently in use. Clinicians must deal with a plethora of modes and parameters that must be carefully considered and monitored throughout the ventilation process. The mismanagement of all these modes and parameters can cause serious health problems for the patient. A lack of ventilation assistance can cause fatigue in the diaphragm forcing the accessory inspiratory muscles to take over the work of breathing which can lead to respiratory acidosis. On the other hand, if the patient is being over ventilated, the respiratory drive decreases and the patient can develop an unintentional acute respiratory alkalosis [32]. The main reason for this is that positive pressure ventilators are extremely complex machines that require a deep understanding of their mechanism and effects on the patients. This is an ongoing issue due to the lack of communication between engineers and medics.

Negative pressure ventilation on the other hand has a massive advantage over positive pressure ventilation due to not requiring endotracheal intubation or sedation to ventilate the patient, in addition to air not being forced into the lungs. However, it has the drawback of being old technology with an archaic control system and bulky. Not to mention the limited movement of the patient and the restricted access of the clinician to the patient. This limited movement can cause back pains and the negative pressure can cause rib fractures. Besides, negative pressure ventilation can impair sleep quality, and the lack of upper airway protection could result in upper airway obstruction [33].

## Modern negative pressure ventilators

In light of the COVID-19 pandemic, a shortage of ventilators was witnessed in hospitals all around the world, which prompted aspiring engineers to think of a way to provide low-cost ventilators to help save lives. This brought back the topic of negative pressure ventilation into the limelight, due to it being comparatively cost-effective and having lower health complications [34], where previous designs were examined and improved to be able to provide reliable ventilation in a modern way.

### Exovent

**Fig. 6:**
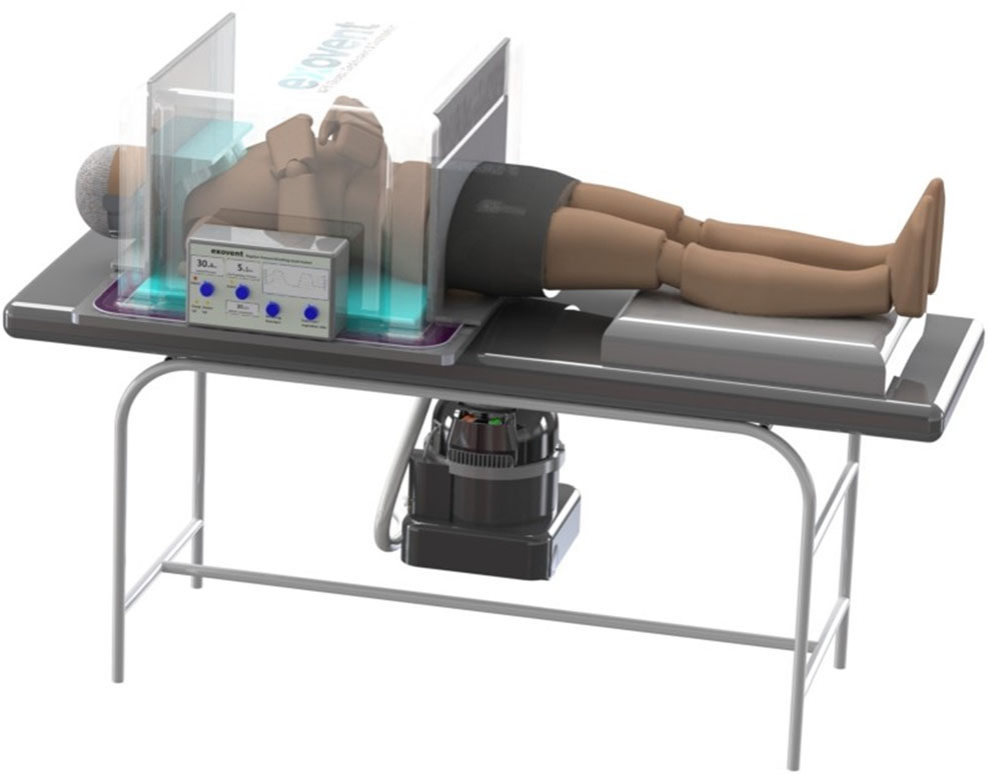
Exovent model [35]

Developed by the University of Warwick, Marshall Aerospace & Defense Group, and other collaborators, Exovent is a breathing support device that uses negative pressure ventilation and whose design is similar to the iron lung [35] (Fig. 6).

The exovent adapts the old technology of negative pressure ventilation that was used in the iron lung, where a pump is used to create negative pressure around the chest and abdomen inside the chamber to aid breathing. Exovent consists of a chest enclosure (patient pressure vessel), a control unit, and a power unit. The tank is a box or half-cylinder, according to their newest design, that encompasses the patient from the neck to the hips, unlike the iron lung which held the patient’s whole body from the neck.

The tank is made of lightweight material that resists a vacuum force of at least 50 mbar, equivalent to 50 cm H_2_O. Since patients are placed inside the tank, the exovent has the advantage of not needing to have several sizes for use for people with different builds as it is made to be spacious enough to allow patients to turn around comfortably inside. The seals for the neck and hips are made of a material that prevents leakage while also not irritating the skin where it touches.

The control unit consists of a controller that collects information from pressure sensors placed in the tank and the pump, which controls the speed of the pump via a power controller (TRIAC) in addition to controlling a valve connected to the enclosure to manage the air intake. This allows for the I:E ratio and respiratory rate to be controlled and allows the exovent to support different configurations. These configurations include one where the vacuum level is controlled by the speed of the pump, one where the pump is run at a constant speed and the vacuum is controlled by the air intake valve and one where the vacuum is controlled by the air intake throttle between the enclosure and pump with the pump run at a constant speed. Moreover, the pump, which represents the power unit, is reported to allow a maximum negative pressure of 50mbar and a minimum airflow of 20 L/s, in addition to decreasing the vacuum pressure to −30mbar in less than 0.5 s.

### Hayek biphasic cuirass ventilators (BCV*)*

**Fig. 7:**
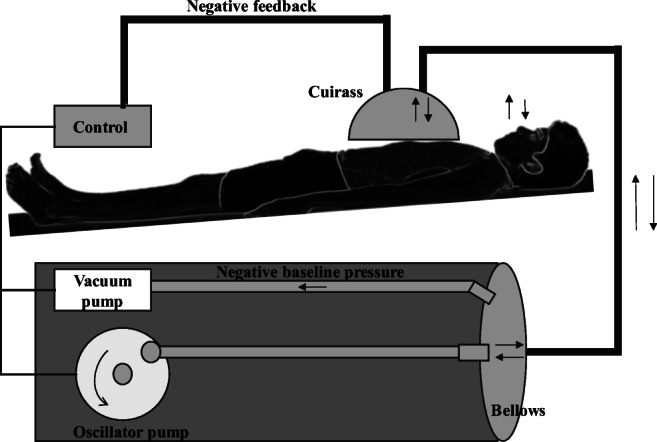
The Hayek Oscillator (HO) [36]

Dr. Zamir Hayek, the leading pioneer in cuirass ventilation, has developed the Hayek Oscillator (HO) and up to the current RTX ventilator series. All his products are manufactured and distributed by Hayek Medical, a United Hayek Industries division [36]. HO had 3 main units: the cuirass, the power unit, and the control unit. The cuirass is a chest enclosure that covers the patient’s thorax beginning at the upper chest and ending at the upper abdomen. The material used for the cuirass was transparent lightweight flexible plastic. To fit on the patient properly, the cuirass was edged with a seal made of foam. A wide bore tubing connected the cuirass to the power unit, as shown in Fig. 7, which consisted of a diaphragmatic pump that has a maximum stroke of 0.5 L and can operate over 8 to 999 cycles per minute (CPM) where the generated pressures oscillate around these frequencies. Within the power unit was another pump which set a negative baseline that frequencies oscillate about, providing lung volume control. The performance of the power unit is adjusted by negative feedback from a pressure transducer, attached to the interior of the cuirass. This automatic control unit allows setting the frequency, inspiratory and expiratory pressures, and I:E ratio [37].

Many articles were written regarding the use of HO in different situations, especially during microlaryngeal surgeries, and discussed many advantages of HO [38–40]. Firstly, HO did not require endotracheal or tracheostomy intubation which eliminated complications associated with intubation and allowed a completely unobstructed view for surgeons. Although HO did not provide airway protection at the level of cuffed tracheal tubes, it was able to blow any debris out of the airway by adjusting the I:E ratio to make expiration more rapid and thus more forceful than inspiration. With the low tidal volumes it provided, movements of the larynx were extremely less and appeared to be minimum at a frequency of 120 CPM and I:E ratio of 1:1. In the case of any disruption, the oscillator can be easily stopped without affecting the patient in a period of 2 to 4 min. Unlike invasive positive pressure ventilation, OH improved venous return, and the overall cardiac output due to the mean thoracic pressure is provided [40].

The recent RTX ventilator series is meant to provide safe non-invasive complete ventilation that is easily used by clinicians and patients. The latest version is the MRTX, a user-friendly rugged portable BCV. It is a compact system with dimensions of 14×14×18 cm and a weight of 2.5 kg instead of the diaphragmatic pump used in HO. The pumping system consists of a turbine that pushes gas between 2 disk valves, which act as pressure regulators, to generate oscillations over a wide frequency range of 6 to 1200 CPM and pressures of −50 to +50 cm H_2_O. A digital pressure sensor was replaced in the pressure transducer in the control unit. The respirator is powered by a battery of 1 kg that can stand up to 4 h. MRTX cuirass is very similar to that of HO. However, in the new version, Velcro tapes are used to secure the cuirass over the patient’s thorax and the tubing that connects the cuirass to the power unit is made 22 mm wide. Now, there are 12 different sizes, according to the patient’s weight, of the cuirass provided by Hayek Medical. However, the cuirass faced severe criticism in terms of permitting efficient ventilation, while not supporting diaphragmatic contractions properly [40].

## Proposed vessel structure

Although the cuirass is more appealing than the tank due to fact that it allowed more freedom to the patient and more accessibility for the clinician in addition to being transparent and lightweight, it is much less efficient than the tank version. This is because the cuirass ends at the patient’s upper abdomen providing ill diaphragmatic contractions and thus ill ventilation [41], where the tank design proves to be superior as it covers the entire abdomen to the hips.

This brings the discussion to find the most suitable structure and material for the vessel, which must be found by comparing other potential structures and materials and simulating their behavior. Since it was decided that the cuirass is inefficient, only the tank enclosures will be considered. The tank ventilator can be semi-cylindrical or prismatic, where each one will be discussed below in detail.

### Semi-cylindrical

Cylindrical pressure vessels are widely used in the oil and gas industry as they can, for instance, store liquified gases that are of high pressure. This is because the circular shape is simple and robust, making it the most efficient structural form. Yet, when compared to prismatic pressure vessels, cylindrical pressure vessels have their advantages and disadvantages, depending on what the vessel is required to do. A semi-cylinder will be discussed here due to space limitations in medical environments [42].

The principle stresses of the can be estimated as in Eqs. (1) and (2).

1 $$ {\sigma}_{\mathrm{axial}}=\frac{P\cdotp r}{2t} $$

2 $$ {\sigma}_{\mathrm{hoop}}=\frac{P\cdotp r}{t} $$

where *P* is the internal pressure in pascal, *r* is the inner radius in meters, and *t* is the thickness in meters. Hoop stress is double the axial stress and, thus, determines the required thickness of the vessel [43].

**Fig. 8:**
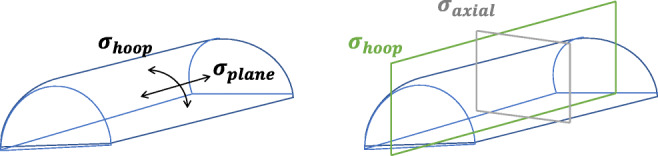
Surface stresses of the main body of a semi-cylindrical vessel

The stresses working on a semi-cylindrical vessel can be deduced through analyzing the static equilibrium state, where the net force is equal to zero. A semi-cylindrical pressure vessel mainly witnesses membrane principle stresses including axial stress, along the *y*-axis, and hoop stress, along the circumferential axis in the tangential direction, as shown in Fig. 8 [43].

**Fig. 9:**
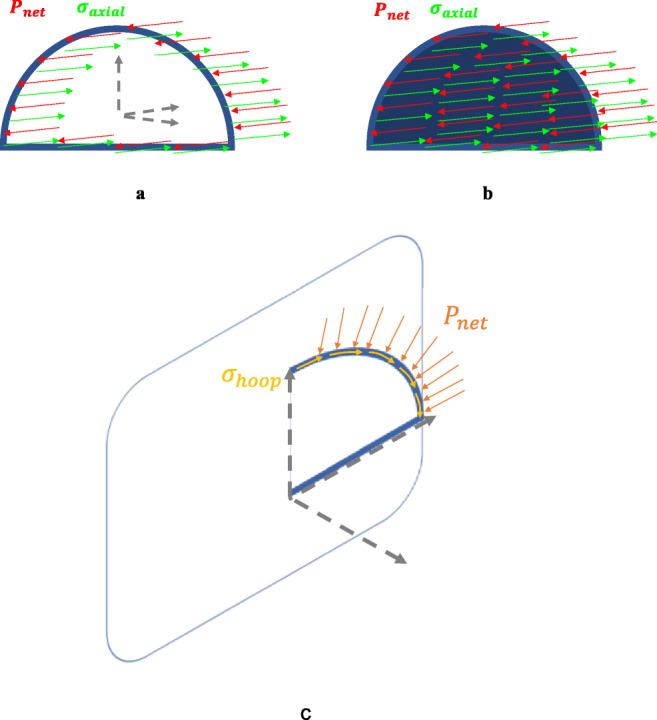
Net pressure (red arrows) and axial stress distribution (green arrows) at **a** the middle and **b** end of the vessel and **c** net pressure and hoop stress distribution throughout the vessel

Figure 9 shows the pressure forces acting on the vessel and the corresponding resisting forces which yield axial stress and hoop stress, respectively. Since the vessel is expected to be thin-walled, where its inner radius to its thickness ratio is greater than 10, radial stress can be ignored. For simplicity, the rectangular base of the vessel is not encountered in the stress analysis, as it will be considered a fixed point in the structure. This is because it is supported by the bed and the patient’s weight.

As mentioned earlier, the pressure vessel is meant to provide the patient with enough space to lie in while not occupying a huge space from the room. Since the vessel is thin-walled, both the inner space of the vessel and the overall space that it occupies from the room can be estimated by calculating its capacity, which is the volume of the structure if it was completely full and not hollow. A filled cylinder has a capacity, *V*_C − Max_ , calculated using Eq. (3).

3 $$ {V}_{\mathrm{C}-\operatorname{Max}}={A}_{\mathrm{Base}}\cdotp h=\pi\ {r}^2\cdotp h $$

where *r* is the inner radius and *h* is the length of the cylinder. Thus, a filled semi-cylinder would have half of that capacity, *V*_SC − Max_, giving the final Eq. (4).

4 $$ {V}_{\mathrm{SC}-\operatorname{Max}}=\frac{V_{\mathrm{C}-\operatorname{Max}}}{2}=\frac{\pi {r}^2\cdotp h\kern0.5em }{2} $$

The vessel is expected to fit on a hospital bed. Thus, its diameter must be equal to or less than the width of the bed. Therefore, as per TotalCare ICU bed dimensions, the expected radius is

$$ r=\frac{d}{2}=\frac{924\ \mathrm{m}\mathrm{m}\ }{2}=462\ \mathrm{m}\mathrm{m}=0.462\ \mathrm{m} $$

The length of the vessel must be equivalent to or smaller than the upper adjustable part of the bed which has a maximum value of 850 mm (0.85 m). Therefore, the capacity that a negative pressure ventilator semi-cylindrical vessel has, using Eq. (4), is

$$ {V}_{\mathrm{SC}-\operatorname{Max}}=\frac{\pi\ {r}^2\cdotp h\ }{2}=\frac{\pi {(0.462)}^2\cdotp (0.85)\ }{2}=0.285\ {\mathrm{m}}^3 $$

It is important to note that since the vessel covers the patient’s body from the neck, two seals, one at the neck and the other at the torso, are required to eliminate air leakage and preserve the vacuum inside the vessel.

### Rectangular prism

Prismatic pressure vessels are not very common. This is mainly because their sharp edges and right angles enormously contribute to their increased stress.

**Fig. 10:**
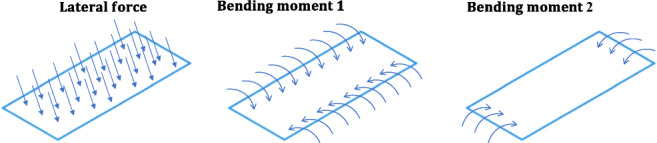
Prismatic pressure vessel lateral force and mending moments

Unlike cylindrical pressure vessels, membrane forces of a prismatic pressure vessel are not constant due to the bending moments that arise from the forces acting on the edges of each plate. Therefore, the total stress at prismatic pressure vessels can be estimated as the sum of the bending stresses and the membrane stresses. It is estimated to avoid complicated calculations, the simplest total stress is the lateral pressure load and two-moment conditions for each plate of the prism using the superposition method, as shown in Fig. 10 [42].

**Fig. 11:**
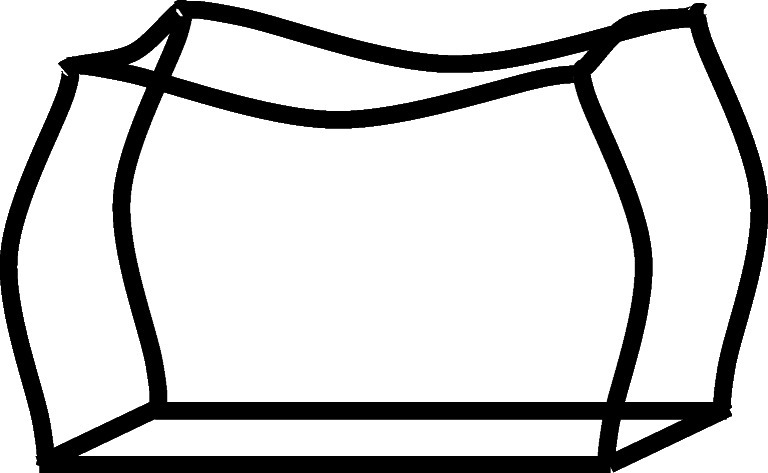
Deformed prismatic pressure vessel

The bending moments have a huge impact on the overall stress which can sometimes yield 20 times more stress than cylindrical stress. The bending moments are greater on longer sides (Bending Moment 1 in Fig. 10) than on shorter sides (Bending Moment 2 in Fig. 10), making it bend first when exposed to external pressure. That will cause the longer sides to bend in and the short sides to bend out, as shown in Fig. 11 [42].

However, the deformation and bending can be significantly reduced by adding support to the structure such as side supports and girders [42]. On the other side, prismatic pressure vessels can have up to 50% better volume efficiency. The capacity of a rectangular pressure vessel, *V*_Rec − Max_, is found using Eq. (5).

5 $$ {V}_{\mathrm{Rec}-\operatorname{Max}}={A}_{\mathrm{Base}}\cdotp h=L\cdotp w\cdotp h $$

where *L* and *W* are the length and width of the baseplate, respectively, and *h* is the height of the vessel, all in meters. Using the same dimensions of the semi-cylindrical vessel, the prismatic capacity is found as follows.

$$ {V}_{\mathrm{Rec}-\operatorname{Max}}=L\cdotp w\cdotp h=(0.85)\cdotp (0.942)\cdotp (0.462)=0.367\ {\mathrm{m}}^3 $$

## Vessel materials

The selection of the vessel material depends on their properties that satisfy the requirements of the system. Materials need to be determined for both the vessel and seals used to seal the neck and torso.

### Main body

In terms of mechanics, the three main properties that determine the chosen materials are rigidity, elasticity, and toughness measured, respectively, by the yield strength, Young’s modulus (*Y*), and the area under a stress-strain curve.

To determine the materials that will be considered to manufacture the ventilator, it is important to recognize the requirements of the system. Given that the vessel will be subjected to a high amount of pressure, it is imperative that the material has to be strong enough not to collapse or have permanent deformation due to the applied negative pressure. Besides, the vessel should be clear or transparent to enable the clinician to observe the patient’s chest and abdomen movement, which enables the detection of patient deterioration when the respiration rate (RR) and chest movement change. The weight of the vessel is also an important factor to consider given that the iron lung was abandoned precisely because it was too bulky and heavy. Finally, the material must also be safe for humans to touch and interact with.

Considering the given requirements, it can be concluded that the most suitable materials are plastics which will allow the ventilator to be lighter than if metal was used, in addition to being non-reactive, poor conductors, strong, and durable. Then, following the requirement of biocompatibility, transparency, and tensile strength, the materials can be further narrowed down to acrylic and polycarbonate.

The comparison is made in Table 1 between acrylic and polycarbonate in terms of properties.

**Table 1:** Comparison between vessel materials

Selection criteria	Materials	
	Acrylic	Polycarbonate
Tensile yield stress	75 MPa	70.0 MPa
Young’s modulus	3.3 GPa	2.4 GPa
Max compressive stress	> 110 MPa	> 80 MPa
Biocompatibility	Biocompatible	Biocompatible
Conductivity	Conductive (must be spray coated)	Insulative
Transparency	> 92%	88–89%
Density	1.19 2 g/cm^3^	1.2 g/cm^3^

Acrylic and polycarbonate are both suitable in terms of their properties shown in Table 1. However, when drilling or putting it under impact, acrylic cracks, but polycarbonate does not. This is because polycarbonate has an impact resistance 250 times higher than acrylic.

### Seals

The selection of material dedicated to seals is very essential because it will determine the leakage from the system. Seals must be extremely elastic and flexible. Nevertheless, they need to be biocompatible and not cause any skin problems to the patient.

When it comes to sealing, rubber always comes to mind. However, rubber has types that can be used in contact with the human while the other cannot. Two commonly used rubber types are neoprene, used in wetsuits, and latex, used in gloves and swim caps.

From Table 2, it can be seen that latex can tolerate pressure more than neoprene. However, it is not selected to be used because it can cause allergies to some people.

**Table 2:** Comparison between seal materials

Selection criteria	Materials	
	Neoprene rubber	Latex rubber
Density	0.192 g/cm^3^	1.15 g/cm^3^
Tensile yield stress	> 1.38 MPa	17 MPa
Elongation at break	400%	500%
Biocompatibility	Completely biocompatible	Can cause allergy to some people

## Modeling and simulation

Since the stresses of a prismatic vessel were not specified due to calculation complexity, simulating the pressure effect on the structures using ANSYS is very helpful to determine which structure is better and if the material thickness is suitable, in addition to observing the behavior of each structure with different materials. ANSYS is a popular software used for finite element analysis, where the user can simulate structures or components to find their strength, elasticity, toughness, fluid flow, etc. The number of elements used in both structures was 5873 and 6831 elements to build the semi-cylinder and the rectangular prism, respectively.

ANSYS does not understand the concept of vacuum unless the entire system is simulated to create the outer airflow. Therefore, a ramped external pressure, 50mbar or 5 kPa, was applied to the structures to create the same effect. The material used for both structures is polycarbonate of thickness 4 mm, which will work properly and yield minimum deformation. The dimensions of both structures were made similar, *d*=0.85m, *r*= 0.425m, and *h*=0.85m for the semi-cylinder and *W*=0.85m, *h*=0.425m, and *L*=0.85m for the rectangular prism. In all simulations, the bottom plate is considered a fixed point, the operating temperature is 22 °C, and the material used is polycarbonate where the seals are not considered.

For each structure, the physical outputs of the solid vessels were found as they will determine the amount of space each occupies, the amount of space each provides for the patient, and its portability. Besides, the total deformation and equivalent stress for several simulation cases were considered, which include results for a fixed base in addition to the worst-case scenario of leakage by opening the neck and hips sides of each vessel. The fixed base scenario is more realistic than a fixed corners scenario as the base of the ventilator vessel is fixed to the bed and laid on by the patient.

### Simulation results—semi-cylindrical vessel

The static structural analysis of both structures provided the total deformation, equivalent stress, shear stress, and equivalent elastic strain of each structure. The concern is with total deformation, which shows the deformation throughout the entire structure using a color-coded ascending scale, and the equivalent stress, which tells if the structure deformation is elastic or permanent by comparing it to the yield strength of polycarbonate (around 70MPa).

The physical properties of the structure yielded by the software are shown in Table 3, where the mass of the structure is around 10kg.

**Table 3:** ANSYS: physical properties of the semi-cylindrical vessel

Physical quantity	Value
Volume	9.7061e-003 m^3^
Mass	11.647 kg

**Fig. 12:**
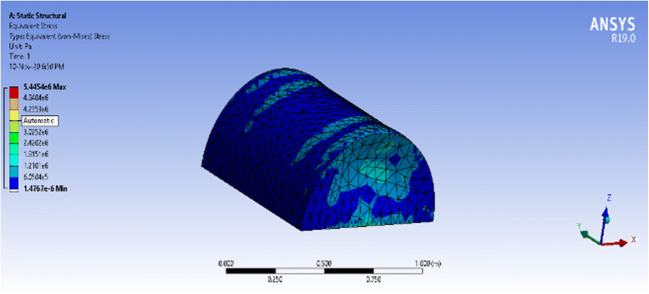
ANSYS simulation of a closed semi-cylindrical pressure vessel of equivalent stress with a fixed base

**Fig. 13:**
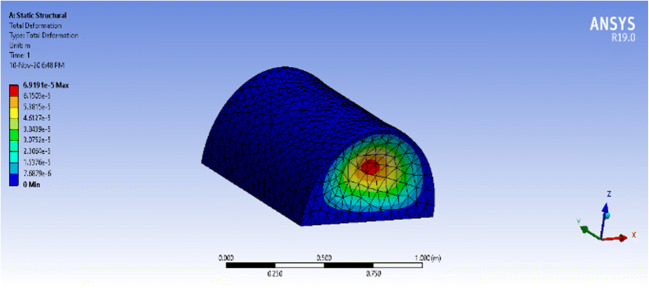
ANSYS simulation of a closed semi-cylindrical pressure vessel of total deformation with a fixed base

The maximum equivalent stress that the vessel witnessed with a fixed base was 5.445 MPa which is extremely below the yield stress of polycarbonate, 70 MPa, which means that the vessel will not face a permanent deformation. On the other hand, the maximum total deformation that the vessel witnessed at the center of the frontal, shown as a red circle, is 0.07mm. Thus, the deformation percentage requires 0.07mm/4mm = 1.75% of the material thickness. The simulation results of the equivalent stress and total deformation can be seen in Figs. 12 and 13, respectively, for the closed vessel with a fixed base.

**Fig. 14:**
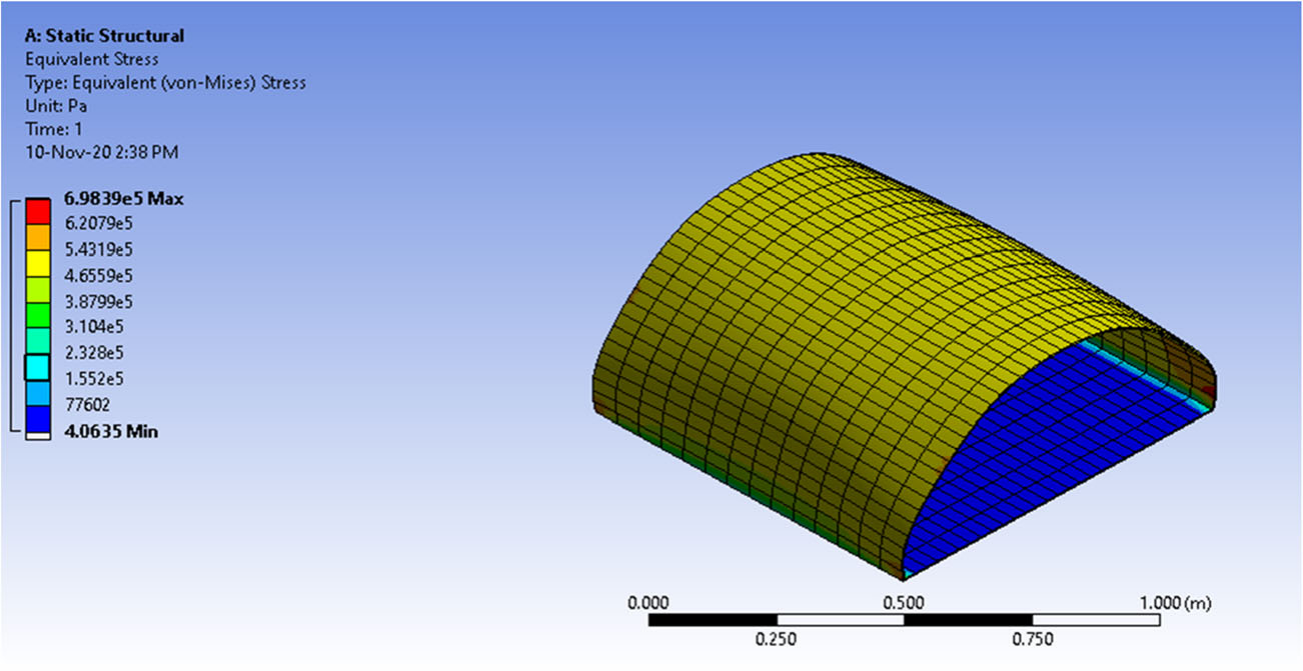
ANSYS simulation of an open semi-cylindrical pressure vessel of equivalent stress with a fixed base

**Fig. 15:**
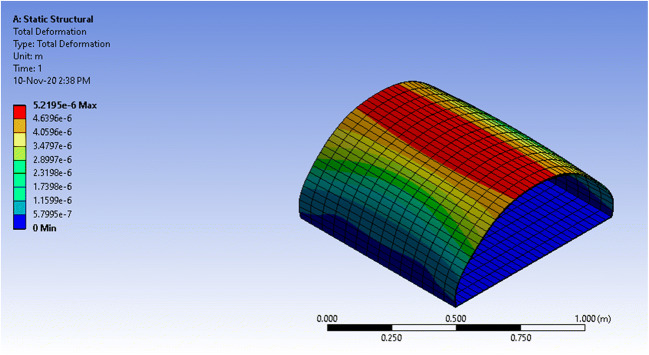
ANSYS simulation of an open semi-cylindrical pressure vessel of total deformation with a fixed base

The worst possible scenario of leakage produced equivalent stress of 0.698 MPa and total deformation of 5.22μm, which are much lower than those produced by a closed vessel, ensuring that the deformation will not be permanent. The deformation and equivalent stress can be seen in Figs. 14 and 15, respectively.

### Simulation results—rectangular prism

The volume of the prismatic vessel is almost double that of the semi-cylindrical vessel. The mass of the structure doubles as well, as can be seen in Table 4, which is undesirable.

**Table 4:** ANSYS: physical properties of the prismatic vessel

Physical quantity	Value
Volume	1.7462e-002 m^3^
Mass	20.955 kg

**Fig 16:**
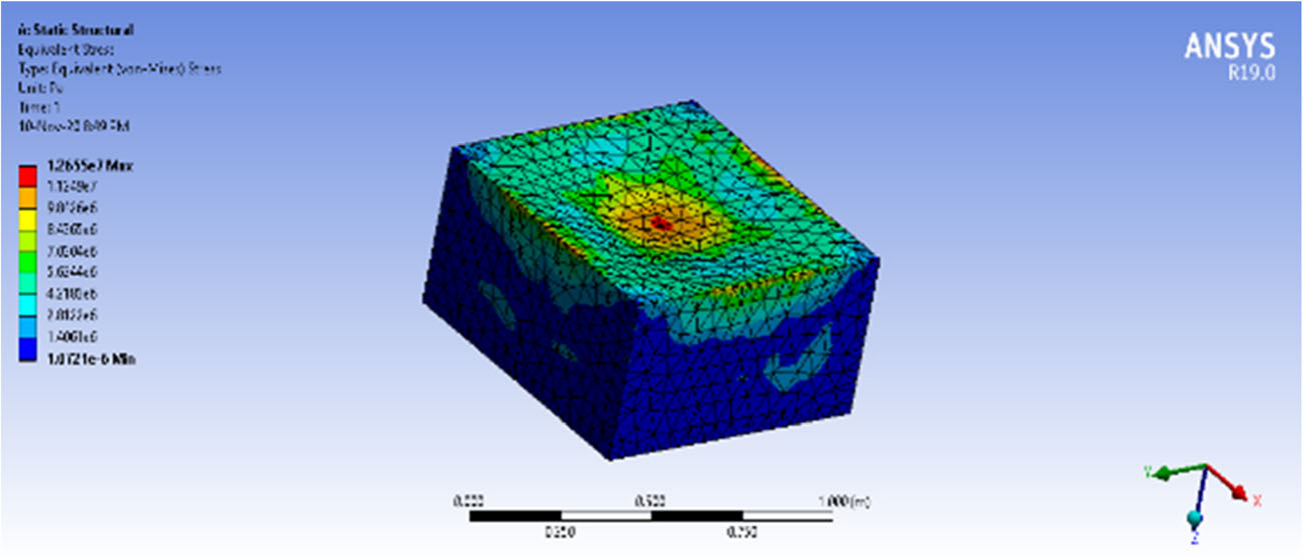
ANSYS simulation of a closed prismatic pressure vessel for equivalent stress with a fixed base

**Fig. 17:**
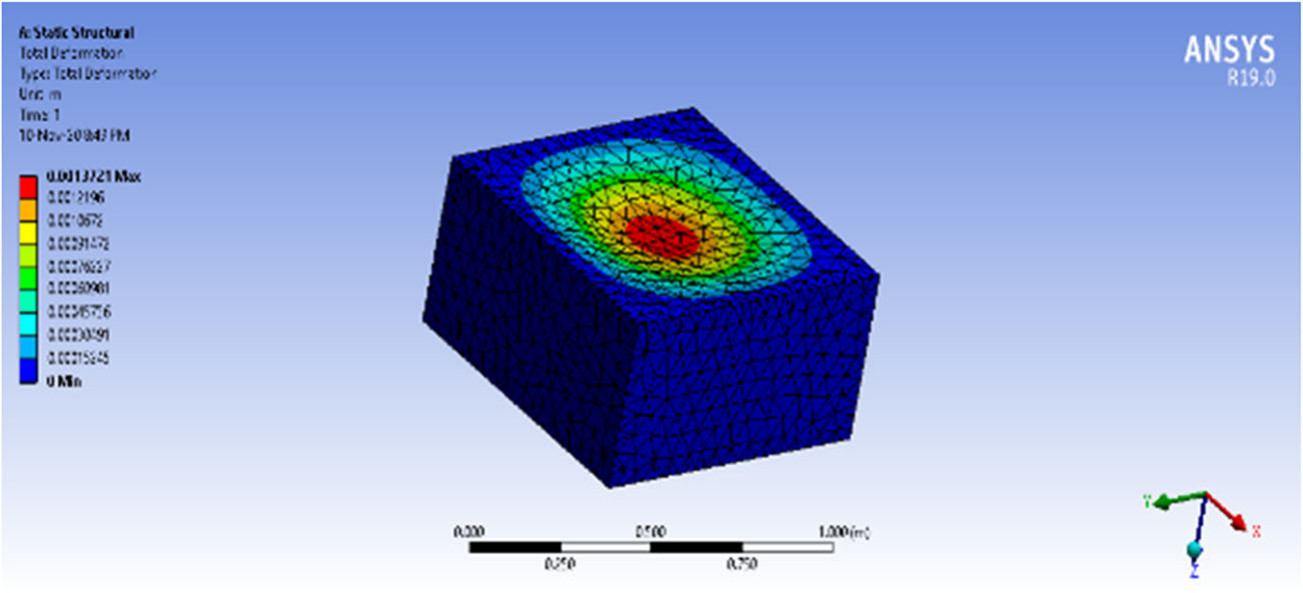
ANSYS simulation of a closed prismatic pressure vessel for total deformation with a fixed base

The vessel witnessed maximum equivalent stress of 12.66MPa which is almost 3 times greater than that of the semi-cylindrical. However, it is still below the yield stress of polycarbonate, 70 MPa, which means that the vessel will not face a permanent deformation. On the other hand, the maximum total deformation that the vessel witnessed at the center of the top plate, shown as a red circle, was 1.37mm. Thus, the deformation percentage is 1.37mm/4mm = 34.25% of the material thickness. The simulation results for equivalent stress and total deformation are seen in Figs. 16 and 17, respectively.

**Fig. 18:**
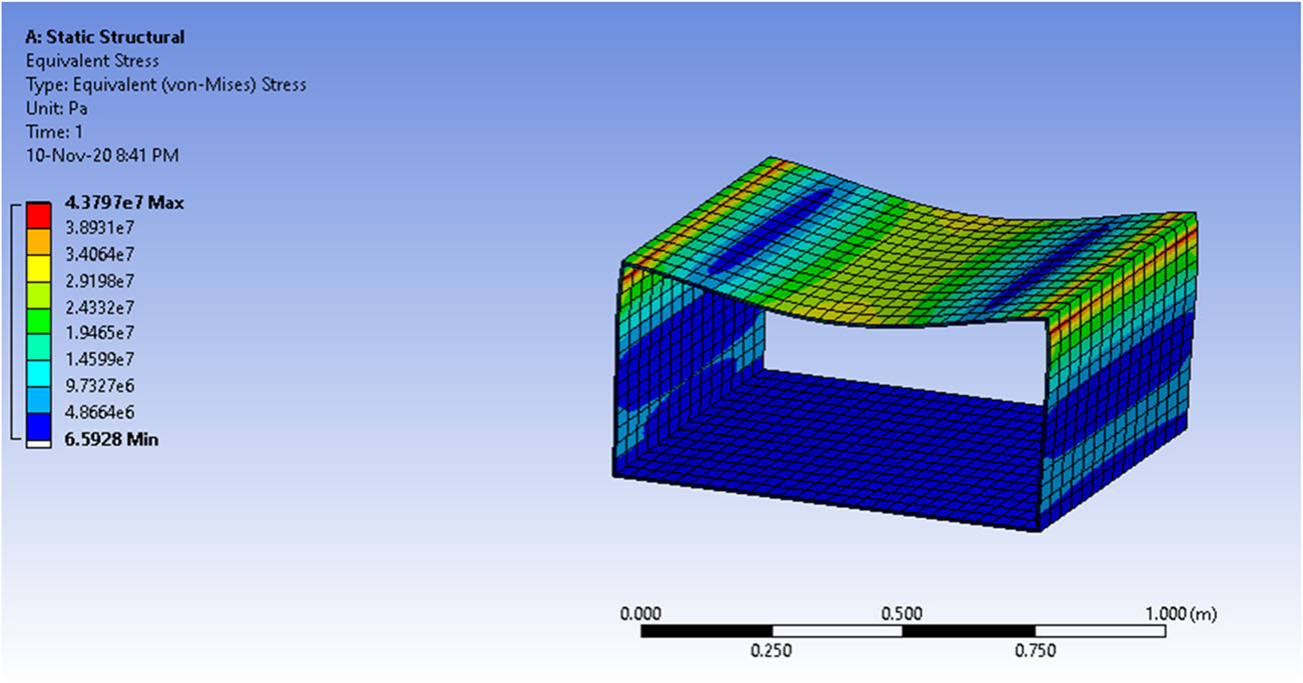
ANSYS simulation of an open prismatic pressure vessel for equivalent stress with a fixed base.

**Fig. 19:**
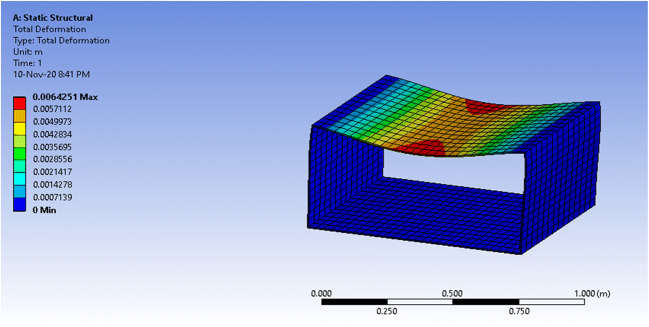
ANSYS simulation of an open prismatic pressure vessel for total deformation with a fixed base

The equivalent stress for an open prismatic vessel is 43.8 MPa and the total deformation is 6.42mm, which is higher than the thickness of the material of 4mm. This results in a deformation percentage of 6.42mm/4mm=160.5%, higher than polycarbonate’s elongation at break, which means that it is most likely to break. The results are shown in Figs. 18 and 19.

**Fig. 20:**
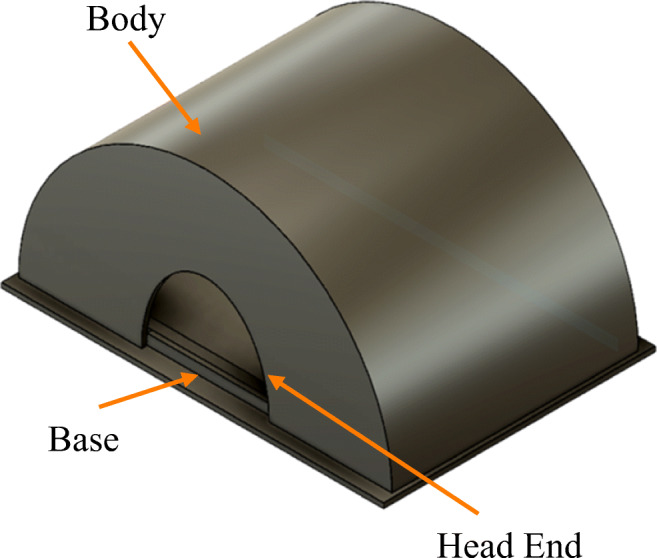
Final design of the proposed system

From the simulations, it can be concluded that both structures can operate under the maximum pressure condition without major failures for the normal case scenario. Meanwhile, the worst-case scenario with very high leakage shows that the prismatic vessel performs worse than the half-cylindrical vessel. In summary, the results show that a semi-cylindrical vessel will be a better solution for the patient enclosure for a negative pressure ventilator. The final design of the proposed system is shown in Fig. 20.

## Conclusion

Due to the huge wave of patients during the pandemic, there was a huge stress on the hospitals’ resources in terms of logistics, staff, medical equipment, etc. Medical equipment such as ventilators which helped patients suffering from breathing problems is really demanding during such pandemics. An investigation into a low-cost method of ventilation began due to the shortage of mechanical ventilators in hospitals all around the world with the spread of COVID-19. The old technology of negative pressure ventilation comes in front when the researchers start looking for a low-cost and safe ventilator compared to the current widely used positive pressure ventilators. The evolution of negative pressure ventilators and their eventual replacement by positive pressure ventilators are discussed in this paper, in addition to a comparison between both methods in terms of their advantages and limitations, and complications. The paper also discusses the structure and material for a low-cost negative pressure ventilator and the structure and materials were simulated using ANSYS software. The theoretical and practical results are used to first compare and then select the best structure and material to be used for a low-cost negative pressure ventilator that will ease the burden on the healthcare system and reduce possible complications affecting patients. As mentioned in the introduction, the major contributions of this work can be summarized as (i) briefing the development of mechanical ventilators, (ii) comparing NPV and PPV in terms of cost-effective and lower health complication solution, and (iii) concluding the structure and material that can be used for the patient enclosure of an NPV with the help of simulation results. The authors would like to extend the work with actual working prototype development to confirm the simulation results. The authors also have plans of resolving the limitations which include noise cancellation, upper body access, and fitting seals to different patients, which are currently challenging to the NPV solutions.
